# Assessment of Left Ventricular Shape Index and Eccentricity Index as Promising Parameters for Detection of Left Ventricular Remodeling in Cardiovascular Events

**DOI:** 10.2174/011573403X357558250122062037

**Published:** 2025-02-07

**Authors:** Fatemeh Jalali-Zefrei, Zobin Souri, Faranak Izadi Benam, Paradise Fatehi Shalamzari, Pouya Yektaee, Seyedeh Zohreh Mohagheghi, Aliasghar Tabatabaei Mohammadi, Soghra Farzipour

**Affiliations:** 1 Department of Radiology, School of Medicine, Poursina Hospital, Guilan University of Medical Sciences, Rasht, Iran;; 2 School of Medicine, Tehran University of Medical Sciences, Tehran, Iran;; 3 School of Medicine, Guilan University of Medical Sciences, Rasht, Iran;; 4 School of Medicine, Iran University of Medical Sciences, Tehran, Iran;; 5 School of Medicine, Urmia University of Medical Sciences, Urmia, Iran;; 6 Melorin Biotech, London, UK;; 7 Department of Cardiology, School of Medicine, Cardiovascular Diseases Research Center, Heshmat Hospital, Guilan University of Medical Sciences, Rasht, Iran

**Keywords:** Left ventricular remodeling, shape index, eccentricity index, gated SPECT, cardiovascular disease, cardiac magnetic resonance

## Abstract

Left ventricular remodeling (LVR) refers to the changes in the size, shape, and function of the left ventricle, influenced by mechanical, neurohormonal, and genetic factors. These changes are directly linked to an increased risk of major adverse cardiac events (MACEs). Various parameters are used to assess cardiac geometry across different imaging modalities, with echocardiography being the most commonly employed technique for measuring left ventricular (LV) geometry. However, many echocardiographic evaluations of geometric changes primarily rely on two-dimensional (2D) methods, which overlook the true three-dimensional (3D) characteristics of the LV. While cardiac magnetic resonance (CMR) imaging is considered the gold standard for assessing LV volume, it has limitations, including accessibility issues, challenges in patients with cardiac devices, and longer examination times compared to standard echocardiography. In nuclear medicine, LV geometry can be analyzed using the shape index (SI) and eccentricity index (EI), which measure the sphericity and elongation of the left ventricle. Myocardial perfusion imaging (MPI) using SPECT or PET is inherently a 3D technique, making it particularly effective for accurately and consistently assessing LV size and shape parameters. In this context, LV metrics such as EI and SI can significantly enhance the range of quantitative assessments available through nuclear cardiology techniques, with particular value in identifying early LV remodeling in specific patient groups. This article explores the diagnostic significance of left ventricular geometric indices through various diagnostic methods, highlighting the important role of nuclear cardiology.

## 1 INTRODUCTION

Alterations in left ventricular (LV) geometry, commonly referred to as LV remodeling, are frequently observed in various cardiovascular diseases, including coronary artery disease (CAD), hypertension, and cardiomyopathy. This remodeling process can involve changes in LV size, shape, and wall thickness, which are often associated with increased cardiovascular morbidity and mortality. Additionally, the term encompasses other conditions, such as congestive heart failure (CHF) caused by diabetes mellitus, hypertension,valvular disease, kidney disease, or advanced age. While few studies have examined LV shape as an indicator of heart geometry, most research on the clinical implications of LV remodeling has focused on LV volumes and ejection fraction (EF) [1]. Various imaging modalities, including 3D echocardiography, cardiac MRI, and nuclear cardiology techniques, can provide valuable insights into the patterns and degrees of LV remodeling. Understanding the differences in the remodeling process between ischemic and non-ischemic etiologies is crucial for accurate interpretation of these imaging markers. After a myocardial infarction, the initial remodeling is often characterized by regional changes, with thinning and dilation of the infarcted myocardium. Over time, these regional changes can progress to involve the entire LV, leading to global alterations in size, shape, and function. Techniques like 3D echocardiography and cardiac MRI can assess regional variations in wall thickness, as well as changes in LV volumes, sphericity, and ejection fraction. In contrast, non-ischemic causes of LV remodeling, such as pressure or volume overload conditions, tend to manifest with more uniform, global changes in ventricular morphology. These global changes can be evaluated using various geometric indices, including LV mass, relative wall thickness, and the ratio of LV end-diastolic volume to LV mass. Nuclear cardiology techniques, such as SPECT and PET imaging, can also provide insights into the functional consequences of these remodeling processes. By recognizing the distinct patterns of remodeling associated with ischemic versus non-ischemic etiologies, clinicians can better differentiate the underlying cause and tailor their diagnostic and management strategies accordingly [2-4]. Integrating these imaging markers of LV geometry can enhance risk assessment and guide clinical decision-making for patients suspected of CAD or other cardiovascular disorders. Notably, risk assessment may be improved by LV geometry assessment even in patients whose LVEF is intact. Less is known about the capacity of left ventricular morphology, a proxy for left ventricular remodeling obtained from MRI, to forecast incident cardiovascular disease and its association with risk variables in the general population [5]. A comprehensive understanding of the molecular mechanisms underlying cardiac disease and the remodeling of the ventricles associated with the disease is essential for achieving this goal. Utilizing this indicator across various disease scenarios could lead to a deeper comprehension of the development of the disease and intricate remodeling adjustments [6-8]. In this review paper, we will evaluate the predictive significant importance of LV geometry in patients suspected of having CAD with 3-D echocardiography, CMR and nuclear cardiology techniques with more focus on PET and SPECT procedures.

## 2 CAUSES AND MECHANISMS

Ventricular remodeling, which follows myocardial injury and is clinically exhibited by alterations in size, shape, and function, is defined by a collection of molecular, cellular, and interstitial modifications. Furthermore, the onset and progression of ventricular dysfunction, arrhythmias, and a dismal prognosis are linked to a remodeled ventricle [9-11]. Many studies have indicated a correlation between left ventricular morphology and cardiac function. Specifically, the development of eccentric hypertrophy or myocardial infarction (MI) can cause the morphological change of the LV from elliptical to spherical [12]. There have been reports that certain shape factors, like LV shape index, eccentricity, and elongation, can be used to predict the presence of congestive heart failure, diabetes, and major structural and functional problems in the heart. Two forms of LV remodeling may be identified: maladaptive remodeling resulting from various pathophysiological processes such as ischemic heart disease, cardiomyopathy, hypertension, and valve dysfunctions, and the first type, which is physiological and adaptable during development. Infarcted and distant myocardium undergo a variety of ultrastructural, metabolic, and neutrally driven processes that contribute to the complicated pathophysiology of post-ischemic left ventricular remodeling [13-15]. A series of biochemical intracellular signaling events are triggered by cardiomyocyte necrosis and the consequent increased overload of the surviving fibers, which in turn cause reparative alterations like dilation, hypertrophy, and fibrosis to be initiated and modulated. The infarcted wall dilates and thins during the first phase (within 72 hours), which can lead to aneurysmal distortion or premature rupture and encourage the production of intracavitary thrombus. Following MI, there is an abrupt rise in wall shear stress and chamber dilation that affects both the distant myocardium and the tissue around the necrotic region. Moreover, late LV remodeling (beyond 72 hours) impacts the entire cavity and is linked to time-dependent modifications in the geometry of the heart, such as chamber enlargement and the transition from an elliptic to a spheric shape [16-18].

## 3 LEFT VENTRICULAR SHAPE AND ECCENTRICITY INDEX CALCULATIONS IN DIFFERENT DIAGNOSTIC PROCEDURE

**Fig. (1):**
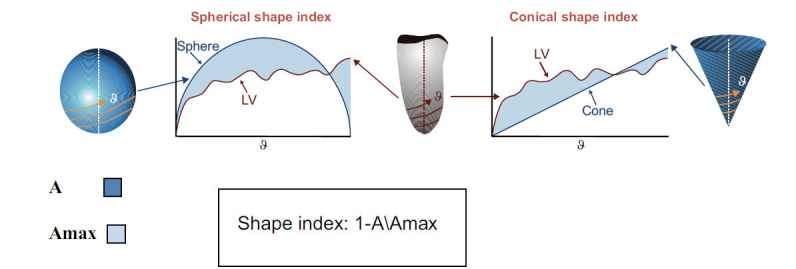
Measurement of LV sphericity in 3-D-echocardiography.

With the advent of 3DE analysis technologies, LV shape has recently attracted more attention, and there is mounting proof that it might include extra diagnostic and prognostic data. Sphericity and conicity, which are global parameters that range from 0 to 1, respectively, indicate how close the ventricle is to a perfect sphere or cone. This was accomplished by taking samples on the 3D LV surface following a helical pattern and comparing the outcome with a signal taken from an idealized reference 3D shape, such as a cone or sphere, using the same process (Fig. **1**) [19, 20].

**Fig. (2):**
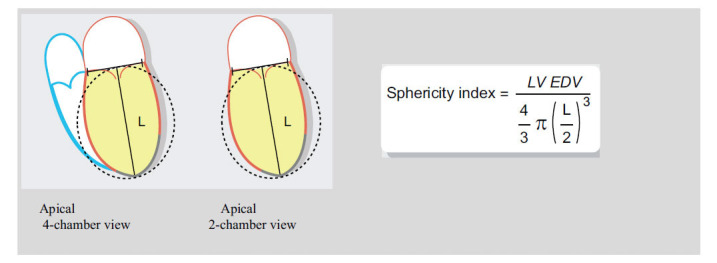
Calculation of of LV spherites index in CMR technique.

The mitral annulus served as the base and the longitudinal axis of the ventricle served as the height of the sphericity index in CMR measurements. The index was defined as the ratio (as a percentage) between the LVEDV and the volume of the sphere at end-diastole [21] (Fig. **2**).

MPI using SPECT or PET is an intrinsically 3D technique and is ideally suited to accurately, reproducibly and automatedly measure parameters of LV size and shape. Fig. (**3**) illustrates the structure of the remodelled heart.

Gimelli *et al*. employed a three-dimensional, global method to measure LV eccentricity (eccentricity index, or EI) [22, 23]. To put it another way, the 3D maximal count mid-myocardial surface of the LV is fitted to an ellipsoid, the major axis of which is b, and the minor axes of which are a and c, are used to compute the index using the following equation:


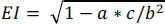


The ellipsoid can be thought of as a “ellipsoid of revolution” obtained by rotating around its major axis if the minor axes are all the same length. The preceding equation may then be simplified as:


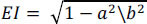


Known also as a spheroid, this kind of ellipsoid is really closer to a sphere the closer an is to b for a perfectly spherical LV, where a = b and EI = 0. The shape index (SI or LVSI), which is another metric for LV eccentricity, is more regional in character. It is calculated as the ratio between the LV cavity's maximal short-axis dimension A and its long-axis dimension B, measured from the endocardial apex to the center of the valve plane [24].


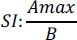


Therefore, when the LV is at its most spherical, local “bulging” can be observed, which will be reflected by a higher SI. This numerical value, in contrast to the EI, will be closer to 1. Although perfusion defects may impact this measurement, the myocardial surface estimation algorithm can maintain surface gradient continuity even in the absence of myocardial uptake, making this concern somewhat mitigated. It is important to note that both the EI and SI can be calculated for ungated images as well as for specific phases of gated acquisitions. In studies examining subgroups with and without LV dysfunction, the end-systolic measurement has shown the strongest correlation with hospitalization for CHF [23, 25].

Gimelli *et al*.'s study likely focused on the ungated EI. However, since all acquisitions were gated, it would be straightforward to extend the analysis to include both end-systolic and end-diastolic frames, possibly utilizing both the EI and SI. Given that SPECT measures myocardial hypoperfusion relative to the LV's maximum uptake region, employing EI or SI as indicators of severe and extensive multivessel CAD would be particularly informative in cases of triple-vessel disease with balanced flow reduction [26, 27]. While the specific CZT SPECT camera used in the study by Gimelli may be capable of overcoming this limitation and measuring coronary flow reserve (CFR) directly through “dynamic acquisition”, this protocol is not typically used in clinical practice, with PET still being the most commonly utilized method today. Nevertheless, other measures such as transient dilatation (TID), LV cavity volumes, and summed perfusion scores could have been beneficial for diagnosing multivessel CAD in a SPECT context. It would have been interesting to explore whether EI or SI provided any additional value over these parameters [28, 29]. As noted earlier, traditionally, LV remodeling has been assessed using echocardiographic methods to evaluate sequential changes in LV geometry, either after a myocardial infarction (MI) or in the context of clinical trials testing new treatments for heart failure and other cardiovascular disorders. Key parameters such as end-diastolic cavity volume, LV myocardial mass, and the ratio of LV myocardial thickness to cavity radius, known as relative wall thickness (RWT), are commonly used in echocardiographic remodeling classifications.

While nuclear cardiology images have relatively limited spatial resolution, making them less suitable for assessing small structures like myocardial thickness and mass, they excel in quantifying LV cavity volumes, TID, shape, and diastolic function. In this respect, LV parameters like the shape index and eccentricity index can enhance the range of quantitative measurements provided by nuclear cardiology techniques. These parameters may also prove useful in the future for specific patient subpopulations (such as diabetics and hypertensives) where early LV remodeling is particularly significant and of great interest [30].

## 4 MEASUREMENT OF LVR WITH DIFFERENT DIAGNOSTIC METHODS

The advent of fast, affordable, and easily accessible echocardiography has significantly enhanced our ability to identify specific left ventricular shapes. Four geometric patterns derived from echocardiography—normal geometry, concentric remodeling, concentric hypertrophy, and eccentric hypertrophy—are proposed to explain the pathophysiologic basis of cardiac remodeling, taking into account factors such as left ventricular mass and wall thickness [31, 32]. Diastolic volume overload on the LV can occur in conditions such as dilated cardiomyopathy, CHD, mitral or aortic regurgitation, and hypertrophic cardiomyopathy. Conversely, systolic pressure overload, caused by hypertension or aortic valve stenosis, often leads to concentric remodeling or hypertrophy of the LV [33]. Regardless of the underlying cause, aberrant left ventricular geometry is considered a valuable echocardiographic phenotype that reflects the degree and persistence of cardiovascular risk factors. This suggests that it may offer more accurate prognostic information than conventional methods. In this context, the impact of LV geometry on clinical prognosis has been extensively studied across various cardiovascular disease (CVD) scenarios [34, 35].

In contrast to MRI and computed tomography, echocardiography is an affordable, radiation-free, and practical method that provides real-time images for the noninvasive assessment of left ventricular geometry and structure in patients with heart disease. However, it is limited by geometric assumptions and foreshortening during image capture, which can result in an inaccurate representation of the true LV shape as influenced by 2D echocardiography. Additionally, 2D echocardiography may be insufficient for detecting subtle variations in global dilation and regional morphology.

The examination of geometric changes in the heart is enhanced by 3D echocardiography, which is closer to the current criterion standard of MRI. This technique can effectively overcome the limitations of 2D echocardiography through the use of a full-volume approach, significantly improving measurement accuracy [36, 37].

The sphericity index (SpI) serves as a valuable metric for quantitatively assessing geometric changes in the left ventricle (LV). It connects the idea of ventricular volume to the remodeling process, which describes how the LV transforms from an elliptical shape to a more spherical one. This concept was first introduced by Tomlinson almost thirty years ago [8]. Using the 3D sphericity index (SpI), Mannaerts *et al*. employed 3D echocardiography to differentiate between patients with acute myocardial infarction (AMI) and those who do not experience subsequent LV remodeling. This parameter has since been applied to various types of LV remodeling. However, despite a substantial amount of published research on the topic, there remains ongoing debate regarding how the SpI changes during LV remodeling [38, 39].

Strong data supports the use of the LV sphericity index obtained from CMR in a range of cardiovascular conditions. According to certain findings, the global and regional LV trabeculation indexes, as well as the LVEF, LV torsion, and mass-to-volume ratio, are all inversely correlated with LV sphericity [40]. Similarly, as dilated cardiomyopathy is closely associated with lower LVEF and increased LV end-systolic volume, the LV sphericity index was able to correctly identify it. Accordingly, the LV sphericity index may be utilized for the risk classification of individuals with heart failure since it is positively correlated with sera levels of the brain natriuretic peptide's N-terminal prohormone [41, 42].

In addition, LV sphericity index is becoming a new tool for prognosticating cardiovascular outcomes. Even with lower LVEF and LGE, the LV sphericity index in patients with dilated cardiomyopathy strongly predicted severe adverse cardiovascular outcomes, such as hospitalization for heart failure, ventricular tachyarrhythmias, and cardiac mortality [43]. Furthermore, in the Nakamori *et al*’s. [44] investigation, the LV sphericity index accurately predicted ventricular tachyarrhythmias in patients with heart failure and lower LVEF, serving as an excellent predictor of appropriate implanted cardioverter defibrillator therapy. However, in apparently healthy individuals, the LV sphericity index may also be helpful in predicting the development of cardiovascular disease [45, 46].

MPI using ECG-gated single-photon SPECT is a more quantitative and reproducible method of assessing myocardial structure because it uses myocardial perfusion tracer counts rather than geometric changes in the myocardium [47]. Research revealed that multivessel CAD was predicted by the left ventricular eccentricity as determined by gated SPECT MPI. Even in healthy persons, there is a strong correlation between the left ventricular shape characteristics and the LV volumes and function in HF patients. The LV shape index has also been used to identify patients with structural and functional cardiac changes brought on by diabetes mellitus who have unfavorable LV remodeling. However, there aren't many studies that have focused on LV shape as determined by gated SPECT MPI in CVD assessments [48, 49].

## 5 DIAGNOSTIC AND PROGNOSTIC VALUE OF LV SHAPE IN DIFFERENT TYPES OF CARDIOVASCULAR EVENTS

In a number of cardiovascular disorders, pathology LV remodeling is linked to an increasing decline in LV function as well as an increase in cardiovascular morbidity and mortality [50, 51]. Conditions with an elevated left ventricular hemodynamic load or those following myocardial infarction, which induce an increase in left ventricular chamber capacity, muscle mass, and fibrous tissue contents, are the most frequent causes. It has been proposed in multiple papers that quantitative LV shape descriptors improve the ability to distinguish between pathological and normal LVs because altered LV eccentricity occurs prior to the apparent change in LV systolic performance [52, 53]. Although left ventricular cardiac geometric indices such as size and sphericity characterize adverse remodeling and have prognostic value in symptomatic patients, little is known about shape distributions in subclinical populations. Gracia *et al*. aimed to measure LV shape variation in a large sample of asymptomatic volunteers and investigate variations between subgroups. 1,991 CMR cases from the Multi-Ethnic Study of Atherosclerosis baseline assessment were used to create an atlas. They concluded that the sphericity volume index (SVI) as an indicator of LV remodeling can predict incident cardiovascular events (CAD, CHD, AF) over 10 years of follow-up in a diverse population [54]. It is unclear if ventricular tachyarrhythmias are linked to echocardiographic remodeling indicators. Yehoshua *et al*. investigated the possibility of a relationship between appropriate implanted cardioverter-defibrillator (ICD) management and a transthoracic echocardiographic (TTE) marker of increased spherical LV remodeling in patients with primary prevention ICDs. The researchers calculated the systolic and diastolic volume (SI) of 278 patients with primary prevention ICDs and 50 controls without structural heart disease or ventricular arrhythmias from TTE images. The SI is defined as the ratio of the biplane LV end-diastolic volume to the volume of a hypothetical sphere with a diameter equal to the LV end-diastolic length. The SI and therapy for ventricular tachyarrhythmias were analyzed.

SI was 0.44 ± 0.02 in healthy, normal adults and 0.65 ± 0.04 in ICD patients (*p*<0.001). For ICD patients with SI in the upper 50% versus lower 50% of SI values, the median time to first appropriate ICD therapy was significantly shorter (1.40 *vs* 2.38 years, *p*=0.02 for conventional ICD patients; 1.54 *vs* 2.65 years, *p=*0.02 for cardiac resynchronization therapy-defibrillator (CRT-D) patients). After multivariable correction, high SI (upper 50%) was independently associated with appropriate ICD therapy (HR 2.2, *p=*0.03 for ICD cohort; HR 4.4, *p=*0.01 for CRT-D cohort). However, SI did not correlate with overall mortality in either cohort [55, 56].

Venkatesh *et al*. investigated the impact of sphericity volume index (SVI), an indicator of left ventricular remodeling, on the prediction of cardiovascular events over a 10-year period in a diverse population. Results showed that LV chamber shape, characterized by sphericity indices, was an independent predictor of CV events. Lower sphericity was a predictor of CVD and CHD, while higher sphericity was an independent predictor of AF. Both extremely low and extremely high sphericity were predictors of HF. The study suggests that lower sphericity may indicate a stiffer ventricle and a stiffer arterial system. This study demonstrated that left ventricular concentric hypertrophy can predict CVD, CHD, and cerebrovascular diseases in large populational studies. LV concentric remodeling is associated with decreased global and regional function, hypertension, and insulin resistance syndrome. Lower sphericity was found to be an independent predictor of CVD and heart failure over a 10-year follow-up period. Higher sphericity was also found to be a strong predictor of heart failure. The study found that LV shape represents the adaptation of myocardial architecture to changes in tissue characteristics and external CV risk factors. However, these indices do not provide additional discrimination ability to conventional CV risk factors and imaging and biomarkers. Advanced modeling techniques that incorporate different aspects of remodeling, such as LV hypertrophy, LV shape, and LV size, may help identify phenotypes with superior prediction and discriminatory abilities. The study is observational and requires further investigation to extend results to populations with prevalent diseases [57].

Another study by Balaban *et al*. devised a technique for quantifying left ventricular (LV) remodeling in non-ischemic dilated cardiomyopathy (DCM), which may be a contributing factor to ventricular arrhythmias and sudden cardiac death. The researchers took end-diastolic cardiac magnetic resonance images from 156 individuals with DCM and late gadolinium enhancement and converted them into three-dimensional LV shape models. They identified patients who experienced composite arrhythmic endpoints of sudden cardiac death, aborted sudden cardiac death, and sustained ventricular tachycardia, by calculating a predictive LV arrhythmic shape (LVAS) score. The best LV shape for estimating the time between arrhythmic episodes was a broad-base, paraboloidal longitudinal profile. The study suggests that biomarkers of LV shape remodeling in DCM can help identify patients at the greatest risk of lethal ventricular arrhythmias [58, 59].

The study by Shaun *et al*. evaluated the use of LVSI as a rapid diagnostic tool for Takotsubo's Syndrome (TS) and Anterior Myocardial Infarction (AMI) in patients without significant left ventricular systolic dysfunction. The study involved 50 TS patients and 50 AMI patients and compared their baseline cardiovascular risk factors, LV mass, and LVEF. The results showed no significant difference in LV mass or LVEF between the two groups. However, there was a significant difference in mean LVSI between TS and AMI. This suggests that LVSI could be a useful diagnostic tool to distinguish between TS and AMI in the acute phase [60].

A meta-analysis of 13 studies involving 1,064 patients found that the utility of the 3-dimensional sphericity index (3D SpI) for evaluating left ventricular remodeling (LVR) remains uncertain. The results showed that 3D SpI was significantly higher in patients with eccentric remodeling compared to the control group. However, no significant difference was found between healthy controls and patients with concentric hypertrophy or myocardial injury. The study suggests that 3D SpI can be used to assess LV remodeling in patients with eccentric remodeling, but it has limitations in predicting concentric hypertrophy and regional or chronic myocardial injury. Overall, the meta-analysis indicates that 3D SpI can be widely used to assess LV remodeling in patients with eccentric remodeling, but its broader utility remains uncertain [36, 61].

Anvari and colleagues investigated the relationship between abnormal LVSI on transthoracic echocardiography and appropriate ICD therapy in patients who received primary ICD implantation. The study included 140 patients, who were categorized into “no ICD therapy” and “ICD therapy” groups. The results showed that patients in the ICD therapy group had LVEF and lower LVSI compared to those without ICD therapy. Notably, an LVSI of ≤1.58 was associated with a fourfold increase in appropriate ICD therapy, even after accounting for LVEF. The study suggests that the simple echocardiographic sphericity dimension index, as a marker of cardiac remodeling, may be an important predictor of appropriate ICD therapy in patients receiving primary prevention ICDs. Furthermore, it could provide additional risk stratification for patients with left ventricular systolic dysfunction [62].

## 6 PROGNOSTIC VALUE OF LEFT VENTRICULAR SHAPE IN NUCLEAR CARDIOLOGY

The term “cardiac remodeling” refers to the series of changes that occur in the left ventricle (LV) following significant injury or damage. These changes lead to substantial alterations in the overall structure and function of the ventricular chamber [63]. The underlying heart disease can influence the origin of the remodeling process. However, the most common causes are acute myocardial necrosis following a heart attack and an unusually high left ventricular workload, as seen in high blood pressure. In these situations, the left ventricle's natural “bullet-like” shape gradually alters to help maintain an adequate stroke volume [64, 65]. Actually, an increased spherical shape of the left ventricle is typically the end result of this harmful remodeling process, which eventually leads to progressive left ventricular contractile failure. Heart SPECT is the gold standard for evaluating left ventricular perfusion. Moreover, contemporary cardiac SPECT scans offer the possibility of obtaining consistent measurements of important structural and functional parameters of the LV. In fact, recent evidence has highlighted the viability and potential clinical utility of assessing both systolic and diastolic functional characteristics during SPECT imaging [66]. In many cardiovascular conditions, LV remodeling is associated with increased morbidity and mortality, along with a gradual decline in heart function. Importantly, assessing LV geometry can enhance risk assessment, even in patients with LVEF [67]. Diabetes mellitus is an independent risk factor for the development of LV remodeling. Ultimately, heart failure results from changes in the structure and function of the LV, which can occur even in the absence of CAD. ECG-gated SPECT is a specialized imaging technique that allows for simultaneous evaluation of myocardial perfusion, left ventricular anatomy, and function. It has been used in various studies to assess the impact of LV shape on overall cardiac performance [68].

The study conducted by Gemelli *et al*. demonstrated that LV eccentricity index (EI), a novel cardiac structural parameter, can be accurately measured during low-dose SPECT imaging with good interobserver repeatability. This allows for the assessment of both the presence and severity of adverse LV remodeling. As a result, LV EI serves as an excellent parameter for the sequential quantitative evaluation of cardiac remodeling, with the coefficient of reproducibility for CZT-derived EI being higher than that of traditional LV functional parameters obtained from gated SPECT. This performance closely mimics the excellent repeatability of measurements from the gold standard, CMR. Specifically, abnormal LV EI, which signifies adverse LV remodeling, was found to be associated with reduced myocardial perfusion and functional parameters. This finding could help identify individuals who are more compromised and may benefit from further evaluation. Notably, there are strong correlations between poorer LV perfusion and functional metrics and lower EI values [28]. The predictive significance of LV geometry indices obtained from gated SPECT in patients with LVEF and normal stress myocardial perfusion imaging was first evaluated by Gaudieri *et al*. Their study found that end-systolic LVSI provided additional prognostic information compared to conventional cardiac risk factors and was an independent predictor of cardiac events within the study population. Thus, measuring LVSI may be important for risk stratification, even in individuals without a history of CAD. The results of this analysis reinforce these findings by showing that, even among patients with normal stress myocardial perfusion imaging, age, diabetes, and end-systolic LVSI are independent predictors of cardiac events during follow-up. Notably, especially in patients with diabetes, event-free survival decreased as end-systolic LVSI worsened. Consequently, higher end-systolic LVSI values increase the likelihood of adverse events, with this risk being greater among those with diabetes compared to those without the condition [69]. Earlier research from the same group also showed that patients with diabetes exhibited abnormal indices of left ventricular (LV) shape, independent of other coronary risk factors and stress-induced myocardial ischemia. While the proportion of diabetic patients with abnormal end-systolic LV sphericity index (LVSI) was approximately twice as high, the overall difference in LVSI between diabetic and non-diabetic patients was relatively small. Additionally, diabetes remained a significant predictor of elevated end-systolic LVSI even after adjusting for other risk factors and demographic characteristics [70, 71].

Nitta *et al*. demonstrated that end-systolic LVSI was substantially correlated with LV volumes and functions in individuals with SSS of ≤3, even though they did not find any signs of a severe perfusion impairment. The ellipsoid shape of the LV progressively gave way to a spherical shape with an increase in LVESV. Adverse LV remodeling most likely stems from an unusually increased LV hemodynamic load, such as arterial hypertension or valvular heart disease, given our analysis excluded patients with severe perfusion abnormalities. The other result showed that end-systolic LVSI rose as LVESV grew and did not grow further when LVESV was greater than 40 ml. It has been suggested that there is a strong correlation between LV shape and cardiac function. According to their findings, the size of the ventricle grows as it transitions from an ellipsoid to a spherical shape in the early stages of the LV remodeling process and continues to grow while it maintains its final spherical shape in the later stages. Patients with continuing LV remodeling may be identified early in life based on their LV shape. Additionally, they discovered a correlation between age and end-systolic LVSI. They demonstrated that LV length decreased with aging in healthy persons, causing LV to become more spherical [72].

Miller *et al*. evaluated the independent predictive value of the eccentricity and shape indices after controlling for significant confounding variables such as LVEF and LV sizes. They found a stepwise increase in MACE rates across the deciles of the shape index and eccentricity index. Furthermore, they discovered that, even after multivariable adjustment, changes in both the SI and EI following stress testing were independently associated with higher MACE rates and provided meaningfully improved patient risk classification. Their findings indicate that changes in ventricular morphology following SPECT MPI should be taken into account when assessing a patient's prognosis, as these measures have significant independent prognostic value [73].

Martinez *et al*. conducted a study that collected data on myocardial hemodynamics, including myocardial blood flow (MBF) at rest, hyperemic MBF during stress, and coronary flow reserve (CFR), as well as LV geometry, encompassing SI measurements at end-diastole and end-systole at rest and during stress, and EI at rest and stress. Using the infarction group as the control, they performed a one-way ANOVA with Dunnett's post-hoc analysis to compare these variables across groups. Their investigation found that the control subjects showed statistically significant differences from all other groups in SI at end-diastole at rest (*p*<0.001) and stress (*p* = 0.005), end-systole at rest (*p* = 0.001) and stress (*p*<0.001), and EI at rest (*p*<0.001) and stress (p<0.001). Furthermore, under stress, all SI and EI values exhibited a linear change that was proportional to the degree of cardiac remodeling. According to their findings, SI and EI measured by 13N-ammonia PET/CT appear to be clinically meaningful variables for the early identification of LV remodeling. However, further research is needed to determine appropriate cut-off values and assess the accuracy of these measures in predicting MACE [74].

Zhao *et al*. sought to investigate whether LVR parameters could provide incremental predictive value beyond the standard perfusion parameter (total perfusion deficit, TPD) from gated SPECT myocardial perfusion imaging (MPI) in patients with known or suspected CAD who had preserved LVEF. Their results showed that patients who experienced MACE had lower EI and higher SI values, as well as higher end-diastolic SI, compared to those who did not have MACE (*p* < 0.05). When TPD was dichotomized using a cutoff of 10%, changes in TPD were also observed between the two groups (*p* = 0.036). Of the overall cohort, 79% had TPD < 10%. MACE occurred in 24.9% of the TPD < 10% group and 33.3% of the TPD ≥ 10% group (*p* < 0.05). After adjustment for clinical and imaging risk factors, diabetes duration, B-type natriuretic peptide, and SI > 0.65 were significant independent predictors of MACE. Importantly, an SI threshold of 0.65 provided incremental prognostic value over the 10% TPD cutoff for risk stratification in this population with preserved LVEF (*p* = 0.031 and *p* = 0.001). These findings suggest that SI and TPD assessed by SPECT have strong abilities to risk-stratify patients with diagnosed or suspected CAD and preserved LVEF, with an SI > 0.65 being a reliable indicator of increased MACE risk [75].

Zhuo and colleagues have skillfully employed GSPECT data from the former VISION-CRT data set to explicate a SPECT-derived eccentricity ESE measure that may offer further significance in the identification of CRT super responders. Their first findings are promising, even if their post hoc analysis has inherent limitations and the therapeutic value of ESE in predicting CRT response is still unknown. The integration of GSPECT desynchrony and LV shape change information into CRT planning and response prediction is yet mostly unexplored field. G-SPECT data seems to be the most dependable and accessible of all the imaging modalities examined in CRT thus far, and it offers early indications that can be used to guide therapy through phase analysis. Therefore, if verified in bigger studies, adding additional metrics like LVEF and LV shape change analysis to phase analysis data may be useful in differentiating patients who respond well to CRT. It's time to see if GSPECT fills in some of the gaps and works with several other complex factors, as mentioned above, to predict CRT response and potentially identify superresponders, even though the authors feel that no single imaging modality can predict CRT response or super response on its own. In order to best predict response, Zhou *et al*.'s work is a step forward in encouraging prospective randomized multicenter investigation of GSPECT parameters in clinical and CRT contexts [76].

## CONCLUSION AND FUTURE PERSPECTIVE

Studies indicate that changes in LV shape and dimensions are crucial for predicting cardiovascular events. Metrics like the Sphericity Volume Index (SVI) and SI serve as independent predictors of conditions such as CAD, HF, and atrial AF. Both low and high sphericity are linked to increased cardiovascular risk. These indices can also enhance the prediction of appropriate treatments for patients with ICDs, especially those with reduced ejection fractions. However, they do not offer additional discrimination beyond traditional risk factors. Advanced modeling methods that incorporate various elements of LV remodeling may improve patient risk assessment. Further research is necessary to confirm these findings in different populations with cardiovascular issues. The integration of SPECT imaging with supplemental evaluations, like ECG-gated SPECT, allows for more comprehensive risk stratification, enhancing the prognostic capabilities for detecting early signs of adverse LV remodeling. Moreover, findings suggest that factors such as diabetes and specific LV geometry can further refine risk assessment and improve patient management strategies. Overall, the evidence emphasizes the importance of monitoring LV geometry alongside traditional risk factors, highlighting the potential for these indices to serve as vital tools in clinical decision-making and predicting outcomes in patients with various cardiac conditions. Future studies should focus on establishing standardized cut-off values for these indices to enhance their clinical applicability. Additionally, the integration of advanced imaging modalities, including multi-modality imaging approaches, may provide deeper insights into LV mechanics and function. Investigating the potential of artificial intelligence and machine learning algorithms to analyze imaging data could further improve the prediction of adverse cardiovascular events and tailor individualized treatment strategies. As we advance, exploring the relationship between LV geometry and patient outcomes across various demographics will be crucial. Collaborations between research institutions and healthcare providers could facilitate large-scale, multicenter studies to validate these findings and promote their implementation in clinical practice. Ultimately, the goal is to develop comprehensive cardiovascular risk models that not only include traditional risk factors but also leverage innovative imaging techniques and emerging biomarkers, thereby enhancing preventive strategies and improving patient outcomes in those at risk for cardiovascular diseases.

## Figures and Tables

**Fig. (3):**
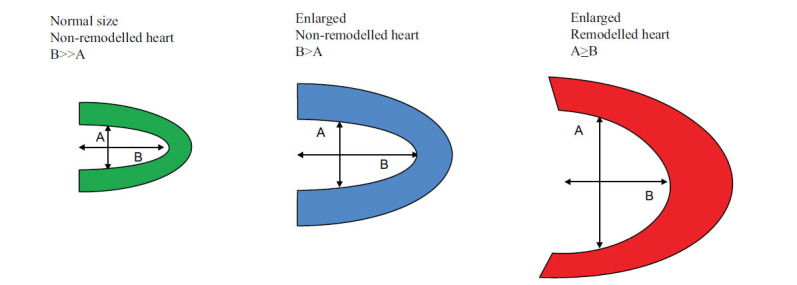
Illustration of remodelled heart. The ellipsoid obtained by rotating the ellipse around b is usually prolate since B>A, but becomes more spherical the closer in length A is to B.

## LIST OF ABBREVIATIONS

CMR: Cardiac Magnetic Resonance
LV: Left Ventricular
LVR: Left Ventricular Remodeling
MACEs: Major Adverse Cardiac Events
SI: Shape Index
2D: Two-Dimensional
